# Study of Optimal Replacement of Thyroxine in the ElDerly (SORTED): protocol for a mixed methods feasibility study to assess the clinical utility of lower dose thyroxine in elderly hypothyroid patients: study protocol for a randomized controlled trial

**DOI:** 10.1186/1745-6215-14-83

**Published:** 2013-03-22

**Authors:** Scott Wilkes, Simon Pearce, Vicky Ryan, Tim Rapley, Lorna Ingoe, Salman Razvi

**Affiliations:** 1 Institute of Health & Society, Newcastle University, Baddiley Clark Building, Richardson Road, Newcastle upon Tyne, NE2 4AX, UK; 2 Institute of Genetic Medicine, International Centre for Life, Central Parkway, Newcastle upon Tyne, NE1 3BZ, UK; 3 Department of Endocrinology, Queen Elizabeth Hospital, Gateshead, NE9 6SX, UK

**Keywords:** Hypothyroidism, Ageing, General practice, Primary health care, Cardiovascular disease, Thyroxine, Feasibility, Randomized controlled trial

## Abstract

**Background:**

The population of the UK is ageing. There is compelling evidence that thyroid stimulating hormone distribution levels increase with age. Currently, in UK clinical practice elderly hypothyroid patients are treated with levothyroxine to lower their thyroid stimulating hormone levels to a standard non-age-related range. Evidence suggests that mortality is negatively associated with thyroid stimulating hormone levels. We report the protocol of a feasibility study working towards a full-scale randomized controlled trial to test whether lower dose levothyroxine has beneficial cardiovascular outcomes in the oldest old.

**Methods/design:**

SORTED is a mixed methods study with three components:

SORTED A: A feasibility study of a dual-center single-blinded randomized controlled trial of elderly hypothyroid patients currently treated with levothyroxine.

Setting: Patients will be recruited from 20 general practices and two hospital trust endocrine units in Northumberland, Tyne and Wear.

Participants: Target recruitment of 50 elderly hypothyroid patients currently treated with levothyroxine, identified in both primary and secondary care settings.

Intervention: Reduced dose of levothyroxine to achieve an elevated serum thyroid stimulating hormone (target range 4.1 to 8.0 mU/L) versus standard levothyroxine replacement (target range 0.4 to 4.0 mU/L).

Randomization: Using random permuted blocks, in a ratio of 1:1, randomization will be carried out by Newcastle Clinical Trials Unit.

Outcomes: Study feasibility (recruitment and retention rates and medication compliance), acceptability of the trial design, assessment of mobility and falls risk, and change in cardiovascular risk factors.

SORTED B: Qualitative study using in-depth interviews to understand patients’ willingness to take part in a randomized controlled trial and participants’ experience of the intervention.

SORTED C: Retrospective cohort study of 400 treated hypothyroid patients aged 80 years or over registered in 2008 in primary care practices, studying their 4-year cardiovascular outcomes to inform the power of SORTED II.

**Discussion:**

This is a study to evaluate the feasibility of conducting a randomized controlled trial in elderly hypothyroid patients in general practice and hospital settings. The results will inform the design of the definitive SORTED II trial to evaluate the effects of lower dose thyroxine in elderly hypothyroid patients.

**Trial registration:**

Current Controlled Trials http://ISRCTN16043724

## Background

The population of the UK is ageing. Over the last 25 years the percentage of the population aged 65 and over increased from 15% in 1984 to 16% in 2009, an increase of 1.7 million people. Over the same period, the percentage of the population aged under 16 decreased from 21% to 19%. This trend is projected to continue. By 2034, 23% of the population is projected to be aged 65 and over compared to 18% aged under 16.

The greatest population increase has been in the number of those aged 85 and over, the ‘oldest old’. In 1984, there were around 660,000 people in the UK aged 85 and over. Since then the numbers have more than doubled reaching 1.4 million in 2009. By 2034 the number of people aged 85 and over is projected to be 2.5 times larger than in 2009, reaching 3.5 million and accounting for 5% of the total population [1].

Thyroid hormones are crucial in controlling metabolism and have an impact on a wide array of tissues including brain, heart, muscle and bones. Thyroid dysfunction is common, affecting all age groups, with a higher frequency in women and older individuals. In the NHANES III survey, a study designed to provide normative estimates of health and nutritional parameters in the USA, a raised serum thyroid stimulating hormone (TSH) level (>4.5 mU/L) was found in 14% of the population aged 80 years or over [2]. In the UK, the population-based Whickham study found that 10% of the population over the age of 75 years had a raised TSH level (>6.0 mU/L) [3]. There is compelling evidence that serum TSH distribution levels increase progressively with age in the reference population with the 97.5th centile being 4.03 mU/L in the 50 to 59 age group and 7.49 mU/L in the 80+ age group [4]. Therefore the prevalence of hypothyroidism and subclinical hypothyroidism is likely to be significantly overestimated in the oldest old and, more importantly, treatment with levothyroxine (LT4) inappropriately initiated in a proportion of these.

Treating elderly patients who have mildly elevated TSH with LT4 does not improve cognitive function [5]. It is recognized that over-treatment with thyroid hormones has the potential for deleterious effects on quality of life, skeletal health, cardiovascular mortality and incidence of atrial fibrillation [6]. This finding has been borne out in the well-designed prospective follow-up study of 85-year-old individuals in Leiden [7]. This study showed that mortality was negatively associated with TSH levels and increased with higher free thyroxine levels. Furthermore, in the same study, subgroup analyses after exclusion of individuals on medication for thyroid disease showed that higher TSH and lower thyroxine levels were associated with reduced worsening of disability, better memory and reduced mortality over 4 years of follow-up. In another study of older untreated adults aged more than 70 years, a mildly elevated TSH between 4.5 and 7.0 mU/L was associated with a slight functional advantage in mobility compared to those with TSH levels of 0.4 to 4.5 mU/L [8]. This suggests that treatment with thyroid hormones could worsen these important outcomes in elderly individuals. Another study of older women (>65 years) found that mortality in thyroid hormone users tended to be higher with a hazard ratio (95% CI) of 1.11 (0.98 to 1.24) and that mildly elevated TSH was not associated with excess cardiovascular mortality [9]. A meta-analysis of observational population-based studies [10], and confirmed by others [11], has shown that mild hypothyroidism in the elderly is not hazardous for vascular mortality and events.

Currently, all individuals with hypothyroidism are treated as a homogenous group irrespective of age with the aim of achieving serum TSH levels within the reference range (0.4 to 4.0 mU/L). However, thyroxine metabolism is altered in advanced age, and ‘age-adjusted’ reference ranges are not employed [12]. Experts suggest that in older hypothyroid patients LT4 treatment should be tailored to have a higher ‘target’ TSH value compared to younger individuals and that ‘prospective therapeutic trials are necessary to clarify the necessity of replacement therapy in the elderly’ [6]. There is neither consensus nor published NICE clinical guidelines to help clinicians manage mild hypothyroidism or subclinical hypothyroidism in older patients. This presents a significant problem for general practitioners (GPs), who manage the majority of these patients [13]. It is imperative to gather evidence and determine whether they should be treated with a more appropriate TSH target, and whether this improves overall as well as cardiovascular morbidity and mortality.

### The future definitive trial: SORTED II

The objective of SORTED II is to address the question of whether elderly hypothyroid patients maintained with a slightly higher TSH reference range, have better morbidity, mortality and cardiovascular outcomes, compared with current usual treatment. Where the feasibility, patient acceptability and parameters to inform a power calculation are lacking for a definitive trial, a well-designed feasibility study is required [14]. In the SORTED study we will test protocol procedures, patient recruitment and retention rates, consent processes, randomization, patient acceptability, and collect data to power SORTED II. This paper describes the three components of the protocol for the feasibility study SORTED, a necessary precursor to SORTED II.

## Methods/design

### Aim: SORTED (A, B and C)

The aim of this study is to explore the design, patient acceptability and required sample size to power the definitive SORTED II trial adequately. Specifically we aim to provide evidence that it is feasible to perform a large randomized controlled trial (RCT) assessing lower dose LT4 in treated elderly hypothyroid patients, evaluating the participants’ experience in the trial as well as morbidity and mortality rates over 4 years in a cohort of patients in general practice.

### SORTED A: RCT feasibility study

#### Aim

The aim is to assess participants’ willingness to enter and complete a single-blinded randomized controlled trial of lower dose thyroxine for elderly hypothyroid patients.

#### Primary objectives

The primary objectives are to show that recruitment to such a trial is possible; to gauge participants’ acceptability of being part of the study; to assess the length of time required to complete recruitment; to assess the dose titration strategy and the length of time required to achieve desired TSH levels; and to gauge medication compliance.

#### Secondary objectives

The secondary objectives are to measure the acceptability and usefulness of generic and validated disease-specific quality of life questionnaires, EQ-5D [15], ThyDQoL and ThySRQ [16]; to assess mobility and risk of falls in this population group as measured by the TUG test and FRAT questionnaire [17]; and to measure changes in specific cardiovascular risk factors including lipid profile, blood pressure and body weight and changes in bone resorption markers.

#### Design

This is a feasibility study for a dual-center single-blinded RCT of elderly hypothyroid patients currently treated with LT4. It is a clinical trial of an investigational medicinal product. The study will compare the standard dose of LT4 (target TSH level 0.4 to 4.0 mU/L) with a lower dose of LT4 (target TSH levels 4.1 to 8.0 mU/L), until TSH is within the desired range. Post randomization, individuals will be assessed at 12 weeks (and LT4 dose adjusted) and at 24 weeks, with a final follow-up phone call at 25 weeks.

#### Study setting and population

Patients will be recruited from 20 research general practices that are supported by the National Institute for Health Research (NIHR), and two hospital trust endocrine units in Northumberland, Tyne and Wear. The study population comprises elderly hypothyroid patients currently treated with LT4.

#### Inclusion criteria

Males and females aged 80 years or over are included if:

• They have been diagnosed with hypothyroidism and treated with LT4 for at least 6 months.

• They are living independently in the community.

• All of their TSH results are within the range 0.4 to 4 mU/L in the 3 months before commencing the study.

• They have provided written informed consent for participation in the study, prior to any study-specific procedures.

#### Exclusion criteria

People are excluded if:

• They have established dementia and therefore deemed incapable of providing informed consent.

• They have other medical conditions, which, in the opinion of the chief investigator, would prevent them from taking part in the study (for example, end stage cancer or severe chronic health conditions where the patient is housebound).

• They are a resident of a nursing home or residential care home.

• They have with thyroid cancer, as they would require high doses of LT4 to suppress their serum TSH.

• They take 25 mcg of LT4 daily: dose reduction will mean that they stop thyroid replacement treatment.

• They do not speak English.

• They have participated in any other investigational trials within the last 3 months.

• They have been prescribed medications that can affect thyroid function (amiodarone, lithium, carbimazole or propylthiouracil).

• They have known or suspected lactose intolerance (this would have implications for the proposed over-encapsulated investigational medicinal product (IMP)).

#### Screening, recruitment and consent

Three methods of recruitment will be adopted for the RCT feasibility study: via a database search of GP practices, opportunistically via secondary care clinics and via poster advertising and self-referral.

**Figure 1:**
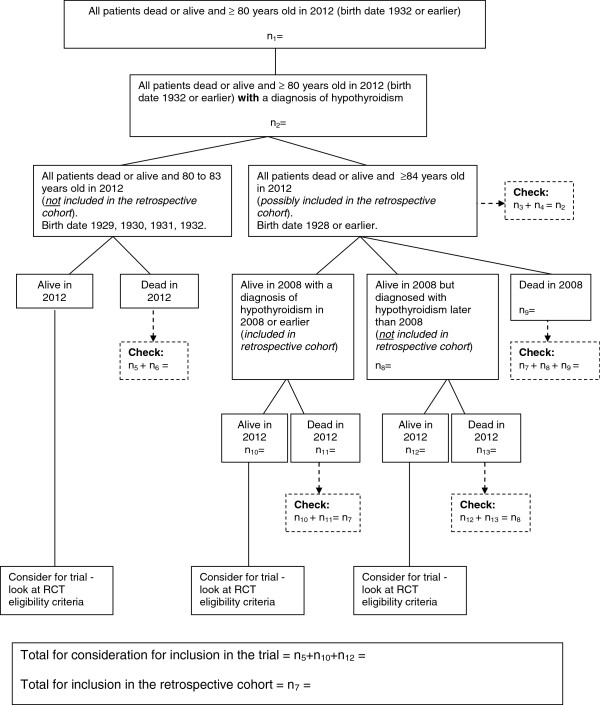
**Search flow chart for the simultaneous retrieval of retrospective cohort data and trial participant data.** For the RCT we need patients with hypothyroidism and who were ≥80 years old in 2012. For the retrospective cohort study we need patients with a diagnosis of hypothyroidism who were ≥80 years old in 2008, and followed up (alive and dead) until 2012. RCT: randomized controlled trial.

Firstly, general practices will act as participant identification centers (PICs) and practice staff will perform a database search using a protocol eligibility criteria flow chart, which simultaneously identifies the SORTED C cohort and potential SORTED A participants (Figure 1). The NIHR Primary Care Research Network team will provide the central study team with a completed eligibility proforma for each potential participant (identified by a unique patient number only). The proforma will be a checklist of all inclusion and exclusion criteria to confirm each participant meets all eligibility criteria. The GP will review the list of eligible SORTED A participants. The practice will approach potential participants by posting a study invitation pack to their home address within 2 working days of the database search.

Secondly, potential participants in secondary care will be identified and invited to participate during routine endocrine clinic visits at the Gateshead Health NHS Foundation Trust and the Newcastle upon Tyne Hospitals NHS Foundation Trust. The initial approach in the clinic will be via the clinician in charge of their routine clinical care. A brief verbal explanation of the study will be provided by the clinician and the participant will be given a study invitation pack.

Finally, we will use study posters in non-research active practices in and around the Northumberland and Tyne and Wear area. Potential participants can contact the central study team directly. The central study team will assess the eligibility criteria then send a study invitation pack.

Potential participants will receive a study invitation pack consisting of:

• Either a pre-prepared invitation letter from the general practice, an invitation letter from their routine clinician in secondary care, or an invitation letter from the central study team in response to a poster enquiry.

• A participant information sheet and consent form.

• A stamped addressed envelope to return the reply slip to the central study team.

On receipt of the reply slip, the central study team will contact potential participants by telephone 7 (+/− 3) days later to confirm the patient’s interest in participating, answer any initial questions and arrange a convenient date and time for the screening visit. If potential participants are not contactable by telephone, then a home visit appointment will be arranged by letter to discuss this information.

If no reply slip is received by the central study team within 4 weeks, a reminder will be sent to the patient’s home address within 2 working days. The reminder will also contain an invitation pack for SORTED B Group 2 (see below) to minimize the number of approaches to each particular patient.

For all potential participants, a screening visit will be arranged either at the patient’s home, the Clinical Research Facility, Leazes Wing, Royal Victoria Infirmary (RVI) or at Bensham Hospital, Gateshead. The central study team (for those patients reviewed at Bensham Hospital or at home) or site-specific research staff (for those patients reviewed at the Clinical Research Facility, RVI) will discuss the study in detail with the patient and answer any queries he/she may have. If the patient wishes to take part in the study, written informed consent will be obtained at this visit.

The eligibility assessments must occur within 3 months prior to the baseline visit and the start of participation in the study. Screening logs will be held securely at the PICs and investigator sites. The screening logs will document details of patients invited to participate in the study.

#### Intervention

Lower dose LT4 achieving a target TSH level of 4.1 to 8.0 mU/L

#### Control

Standard dose LT4 achieving a target TSH level of 0.4 to 4.0 mU/L

#### Randomization

Participants will be randomized to either the usual (current) dose of LT4 (control) or the lower dose of LT4 (intervention), in a ratio of 1:1, using random permuted blocks. Randomization will be stratified by usual (pre-study) LT4 dose (50, 75, 100 and 125 mcg daily). Randomization will be administered centrally via Newcastle Clinical Trials Unit using a secure password-protected web-based system.

#### Blinding

Patients will be blind to treatment allocation. The clinicians will be aware of the LT4 dose so that they can maintain treatment within the specified TSH ranges, either for the usual dose of LT4 (control) or the lower dose of LT4 (intervention), that is, the trial will be single blinded. The research team will be aware of the dose each participant will receive; however, data analysis will be blinded.

At the final visit, the integrity of the blinding will be assessed by asking the participants if they thought they were taking their usual or a lower dose of LT4.

#### Study medication

The LT4 medication, the IMP, will be used within the licensed indications [18] in this study. Nonetheless, since an alternative dosing regime will be tested, additional information is being gathered on safety and efficacy. As manufacture will involve de-blistering/over-encapsulation, the study drug will be treated as an IMP for the purpose of this study, and will be labeled and handled accordingly.

The study drug will be sourced, assembled and packaged by Newcastle Specials (Pharmacy Production Unit) at the Newcastle upon Tyne Hospitals NHS Foundation Trust, MIA(IMP) 17136. The brand of LT4 to be used in this study will be Eltroxin 25 mcg tablets and Eltroxin 50 mcg tablets (Mercury Pharmaceutical Ltd). This will be purchased in blister packs.

The single blind will be achieved by de-blistering and over-encapsulation, using a capsule filler of lactose BP. For doses that are multiples of 50 mcg, we will over-encapsulate Eltroxin 50 mcg tablets; for the remaining 25 mcg, 75 mcg and 125 mcg dose increments, we will over-encapsulate Eltroxin 25 mcg tablets. This ensures the capsules are kept as small as possible for ease of swallowing (DB capsules size A, color: Swedish orange; manufacturer: Capsugel). Capsules will be repackaged into a bottle container (polypropylene) and labeled appropriately.

As it is not feasible to predict in advance exactly how much IMP must be packaged per study dose, predictions will be based on known current doses as per routine clinical practice; production run(s) will generate surplus stock per IMP dose.

The LT4 medication (Eltroxin 25 mcg and 50 mcg) will have a maximum shelf-life of 24 months (less any reduction in expiry due to the shortest expiry date of the blister packs purchased). The IMP will not be stored above 25 °C and will be stored in the original container to protect it from light and moisture, as per the clinical trial label. A temperature log will be maintained as per local pharmacy procedures, and appropriate IMP storage will be guaranteed until the IMP arrives in the patient’s possession.

The side-effect profile of Eltroxin (25 mcg and 50 mcg) is well known and documented [18]. The total daily dose provided to each participant will be between 25 mcg and 150 mcg.

#### Administration of study medication

Participants randomized to the lower dose of LT4 (intervention) to achieve the desired target TSH level of 4.1 to 8.0 mU/L, are likely to have their LT4 medication reduced by 25 mcg once a day at visit 1.

LT4 will be provided as two separate 13-week supplies (dispensed separately at visits 1 and 2; see Table 1). The container will be labeled appropriately but will not indicate the arm of the study to which the participants have been randomized. The label will instead contain a pack number, which will be the link to the relevant packaged dose.

**Table 1:** SORTED A study assessments

	**Pre-screening**	**Screening**	**Baseline**	**Follow-up**		
**Visit**			**1**	**2**	**3**	**4 (phone call only)**
**Assessments**^**a**^	**Within 3 months of baseline visit**	**Week −4 to −1**	**Day 0**	**Week 12 (+/− 7 days)**	**Week 24 (+/− 7 days)**	**Week 25 (+/− 3 days)**
Identification and initial approach (including provision of PIS)	X					
Eligibility criteria checked (as per routine clinical practice)	X					
Written informed consent (including discussion of study in detail, questions answered)		X				
Physical examination (height,^e^ weight, blood pressure, pulse)			X		X	
Venepuncture			X^b^	X^c^	X^d^	
Clinical history (relevant medical history, medication list/history)			X			
Participant-completed questionnaires (ThyDQoL, ThySRQ, EQ-5D)			X		X	
Nurse-administered questionnaires (FRAT, TUG)			X		X	
Randomization (after all eligibility criteria checked and written informed consent obtained)			X			
Study medication prescribed and dispensed (following LT4 dose assessment at visit 2 only)			X	X		
Study medication compliance checks				X	X	
Concomitant medication			X	X	X	
Adverse events				X	X	
Serious adverse events				X	X	X

^a^ Study-specific procedures must only be performed once written informed consent is obtained from the participant; ^b^ 10 ml blood: TSH, FT4, FT3, thyroid peroxidase antibodies, total cholesterol, HDL, triglycerides, serum CTX; ^c^ 5 ml blood: TSH, FT4, FT3 only; ^d^ 10 ml blood: TSH, FT4, FT3, total cholesterol, HDL, triglycerides, serum CTX; ^e^ height to be measured at the baseline visit only.

FT3: free triiodothyronine (thyroid hormone); FT4: free thyroxine; HDL: high-density lipoprotein; serum CTX: serum collagen type-1 cross-linked C-telopeptide; TSH: thyroid stimulating hormone, PIS: participant information sheet.

The study medication will be prescribed by an authorized study physician according to the protocol, using a trial specific prescription (documenting the required IMP dose), and dispensed according to local pharmacy practice. The relevant pharmacy will hold a corresponding list allowing pharmacy staff to correlate IMP pack number with the relevant packaged IMP dose for any particular IMP bottle, thus maintaining the single blind.

To further maintain the single blind, the research team at each site will deliver the trial prescription to the pharmacy and collect the relevant study medication to provide to the patient. Participants will be advised to take the IMP capsule orally once a day as usual. This will be discussed at the time of consent and will be clear in the patient information sheet.

Participants will be informed of potential adverse reactions and advised to contact the relevant study team as required. A study-specific participant contact card will also be provided.

Once randomized, participants will begin their study medication on Day 0 (Table 1).

At 12 weeks (+/− 7 days), serum TSH levels will be checked and the LT4 dose adjusted as follows (the second bottle of IMP should be started at week 12 (+/− 7 days)):

Control (usual) dose arm, TSH level:

≤0.39 mU/L: LT4 reduced by 25 mcg once daily

0.4 to 4.0 mU/L: Stay on current dose of LT4

≥4.1 mU/L: LT4 increased by 25 mcg once daily

Lower dose arm, TSH level:

<0.4 to 4.0 mU/L: LT4 reduced by 25 mcg once daily

4.1 to 8.0 mU/L: Stay on current dose of LT4

>8.1 mU/L: LT4 increased by 25 mcg once daily

The LT4 treatment based on the above regimen will continue for a total of 24 weeks at which point the final set of study-specific assessments will be made (with a further follow-up phone call at 25 weeks). If, during the course of the study, individuals on 25 mcg/day of LT4 require a reduction in their dose of LT4, their LT4 dose will remain unchanged as otherwise they would need to discontinue LT4 therapy completely.

At visit 3, participants on each arm of the study will stop their IMP and will be referred back to their GP, who will promptly prescribe the LT4 dose they were taking prior to study participation. The GP letter (sent once the participant is randomized) and the GP follow-up letter (sent after each participant completes his/her IMP) instruct the GP to check TSH levels for each participant, three months after he/she has completed his/her IMP.

At the end of visits 2 and 3, participants will be asked to return any surplus study drug in the original packaging to the study team, who will verify and document compliance. All unused study medication and packaging will be sent to the local pharmacy for documentation and destruction as per local policy (following appropriate reconciliation by the trial manager).

Documentation of prescribing, dispensing and return of study medication will be maintained for study records.

#### Primary outcome measures

• Participants’ willingness to enter the trial (ratio between those who consented to participate and those who were eligible and approached)

• Participants’ acceptability of study design (as measured by the completion rate of participants in each randomized group).

• Participant recruitment rate (as measured by the number of patients randomized divided by the length of the recruitment period) – the recruitment period runs from the date that recruitment opened to the date of the last randomization.

• Dose titration strategy, described above, and length of time required to achieve desired TSH levels (number of participants in each group that reach target TSH range at both 12 and 24 weeks)

• Medication compliance (tablet count).

#### Secondary outcome measures

• Acceptability and usefulness of three patient-completed questionnaires: a generic quality of life (QoL) questionnaire (EQ-5D), a validated disease-specific QoL questionnaire (ThyDQoL) and the disease-specific hypothyroid symptom checklist (ThySRQ). The time taken to complete the three questionnaires will be recorded and questionnaire completion rates will be calculated. Any third-party help used in a questionnaire’s completion will be recorded.

• Assessment of mobility and risk of falls in this population group as measured by the nurse-administered TUG and FRAT.

• Changes in specific cardiovascular risk factors (lipid profile (total cholesterol, high-density lipoprotein, triglycerides), blood pressure and body weight) and serum collagen type-1 cross-linked C-telopeptide (serum CTX).

#### Data collection and outcome assessments

The following procedures and assessments will be carried out in accordance with the study schedule shown in Table 1.

• Written informed consent.

• Randomization: Usual or reduced dose of LT4

• Participant-completed questionnaires: ThyDQoL, ThySRQ and EQ-5D. A private area will be provided for the participant to complete the questionnaires. If patients refuse to answer certain questions, their wishes will be respected. A record of start and finish times will be retained for patient-completed questionnaires.

• Nurse-administered questionnaires: FRAT and TUG

• Physical examination: Height, weight, blood pressure, pulse.

• Venepuncture: 5 to 10 mL blood (TSH, free thyroxine (FT4), free triiodothyronine (FT3), thyroid peroxidase antibodies, total cholesterol, high-density lipoprotein, triglycerides, CTX

• Clinical history: relevant medical history and medication list.

• Study medication dosing according to dose titration guidelines.

• Study medication compliance and concomitant medication checks.

• Clinical history: adverse events, serious adverse events, changes to concomitant medication.

• Assess integrity of the blind by asking the participant: ‘It is important for the interpretation of the results of the study that we ask you the following: “Do you think you were taking your usual or a lower dose of LT4? Why do you think this?”’

#### Data handling and record keeping

Medical information obtained at each visit will be recorded in the subject’s medical notes or other source documentation in real time. Data will be collected on paper case report forms and entered by an authorized member of the research study team on a secure validated clinical data management system. Data will be entered either at Bensham Hospital (for visits conducted at Bensham or at the participant’s home address) or at the Clinical Research Facility (for visits conducted at the RVI). The clinical data management system will be web-based, allowing access for authorized staff at Newcastle upon Tyne Hospitals NHS Foundation Trust, Gateshead Health NHS Foundation Trust and Newcastle University via password protection. Data will be handled, computerized and stored in accordance with the Data Protection Act 1998. No participant-identifiable data will leave the study site (the case report forms will identify participants by initials, date of birth and unique patient number only). Strict confidentiality will be ensured while dealing with patient-sensitive data in accordance with the Caldicott Guardian’s recommendations (applications will be made to the relevant Caldicott Guardian for use of NHS patient data).

All study data will be held in strict confidence by the investigators and research team. Data and documents will be stored in locked cupboards. A confidential list of trial identifiers and corresponding patient-identifying details will be held at site in a locked cupboard by the principal investigator. The quality and retention of study data will be the responsibility of the chief investigator. All study data will be retained in accordance with the latest Directive on Good Clinical Practice (2005/28/EC) and local policy.

#### Submission of accrual data to the UK Clinical Research Network

This study will apply for adoption to the NIHR Primary Care Research Network Portfolio. Accrual data will be submitted on a monthly basis, by Newcastle Clinical Trials Unit, in accordance with NIHR Clinical Research Network guidelines.

#### Study compliance and withdrawal

Compliance with study medication (IMP) will be assessed and documented by the research team by checking and recording the number of return capsules at each visit. This allows any issues to be addressed immediately with the participant. Compliance will be classed as good if between 80% and 100%.

Participants may withdraw from the study at any time without giving a reason. The investigator may also withdraw patients from the study drug in the event of inter-current illness, adverse events, serious adverse events, suspected unexpected serious adverse reactions, protocol violations or administrative or other reasons. Participants who withdraw will be asked if they would be willing to provide follow-up data collected as per the study protocol. Participants withdrawn from the study will not be replaced.

#### Pharmacovigilance

The safety of the lower dose of LT4 in this study will be evaluated by examining the occurrences of all adverse events, adverse drug reactions, unexpected adverse reactions, serious adverse reactions or suspected unexpected serious adverse reactions as defined by the Medicines for Human Use (Clinical Trials) Regulations [19]. The relationship and expectedness of the adverse event or reaction (causality) will be assessed by the site investigator and relayed to the chief investigator who, on behalf of the sponsor (Newcastle upon Tyne Hospitals NHS Foundation Trust), will notify the regulatory bodies, the Medicines and the Healthcare Products Regulatory Agency (MHRA) and the research ethics committee (REC), within 7 days for fatal and life-threatening events and 15 days for non-life-threatening events. All study investigators will be notified.

Most adverse events and adverse drug reactions that occur in this study, whether they are serious or not, will be expected treatment-related toxicities due to the drugs used in this study. A full summary of product characteristics of Eltroxin have been published [18].

We do not anticipate adverse effects for the lower dose of LT4. Although the current uniform serum TSH reference range of 0.4 to 4.0 is applied across all age groups, in real practice a significant proportion of individuals do have biochemical features of mild under-replacement, which goes unrecognized until a blood test is performed. If severe hypothyroidism develops (TSH >10 mU/L with low FT4 levels), then symptoms including fatigue, weight gain and poor memory may develop. We will assess the clinical impact of thyroid function via hypothyroid-specific symptom checklists, ThySRQ, and biochemically (TSH and FT4 levels) at 12 and 24 weeks to detect individuals who are unwittingly developing severe hypothyroidism. We will adjust their LT4 dose accordingly. Participants will also be provided with a helpline number in case they experience any problems.

#### Statistical analysis

As this is a feasibility study, the analyses of the data collected will be mainly descriptive, with 95% confidence intervals reported where appropriate. As well as patient blinding to treatment allocation, data analysis will also be blinded. At baseline the distribution of all numerical variables will be examined graphically and summarized by appropriate measures of location and spread. Similarly, baseline categorical variables will be tabulated and percentages reported.

#### Sample size calculation

No formal sample size calculation has been performed for the RCT component of this feasibility study as the primary outcome measures are concerned with the recruitment to and randomization of the trial and the acceptability of the trial in this population of patients. As this is a trial with 6 months follow-up, any investigation of changes in key study parameters from baseline to 6 months will only be exploratory. A sample size of *n* = 50 will provide sufficient data [14] to estimate the variability in the responses at baseline and assess the feasibility of the trial.

### SORTED B: qualitative study

#### Aim

The aim is to identify, describe and understand factors associated with participation of elderly people in the trial.

#### Objectives

The objectives are to explore the reasons for participation and non-participation in the RCT (SORTED A) amongst people aged 80 years and over with LT4-treated primary hypothyroidism; to examine the decision-making process of elderly people in choosing whether to participate in the RCT; to explore aspects of health and well-being with differing doses of LT4 replacement for primary hypothyroid disease and to explore issues around retaining elderly people in the RCT.

#### Design and method

In-depth interviews with key informants, 8 to 10 people, who have agreed to participate in the RCT (Group 1) and 6 to 8 people who did not wish to participate in the RCT (Group 2) will be conducted. Participants will be approached sequentially.

To understand participants’ experiences of the trial (*n* = 8 to 10), we will focus on their experience of the medication review (for example, the perceived impact on health and well-being, any fears or concerns and compliance issues) and their participation in the trial (for example, their acceptability of trial processes, changes or improvements they feel we could make and retention issues). We will interview them at approximately 2 weeks after randomization and up to 2 weeks after SORTED A visit 3 or trial termination whichever is sooner. These participants will be recruited to Group 1.

To explore the reasons why patients declined to take part in the RCT, we will focus on their understanding of the trial processes (for example, the information they were given, the encounter with the recruiter and their ideas and/or concerns about randomization and consent) and the intervention (willingness to have medication reviewed and/or changed, ideas and/or concerns about the effect of a dosage change on their health). These one-off interviews will take place within 2 weeks of participants declining to take part in the trial so as to minimize problems of information recall. These participants will be recruited to Group 2.

Participants will have the choice of being interviewed alone or with their significant others, either in their own homes or in an appropriate clinical setting.

#### Participant recruitment and consent

Potential Group 1 participants will be approached by a member of the central study team after they have provided written informed consent for the RCT. They will be provided with a study invitation pack consisting of an invitation letter, a participant information sheet and a consent form for SORTED B Group 1. Following their agreement to participate, the central study team will then contact potential participants by telephone 7 (+/− 3) days later to gauge the participants’ interest in taking part, to answer any initial questions and to arrange a convenient place, date and time for the interview.

Group 2 will be identified when potential participants return reply slips to the central team to indicate that they do not wish to participate in the RCT or when reply slips are not received within the specified 4-week period. This group is not identifiable to the central study team and we will require the relevant NIHR Primary Care Research Network research nurse, the general practice staff or the secondary care clinician responsible for the patients’ routine clinical care to approach this group. Each potential participant will be sent a SORTED B Group 2 invitation pack within 2 working days (after checking living status) consisting of an invitation letter, a participant information sheet, a consent form and a stamped addressed envelope for returning the reply slip to the central study team. For potential participants who do not respond to the initial RCT (SORTED A) invitation, the SORTED B Group 2 invitation pack will accompany the reminder letter for the RCT, minimizing the number of approaches to any particular patient.

For both groups, once initial agreement to participate is given, an interview will be arranged by the central study team either at the patient’s home or at Bensham Hospital. The participant and a friend or relative (if applicable) will be given an opportunity to ask questions. Written informed consent will be obtained.

A pre-screening log will be held by the recruiting GP surgery or hospital site, which will include the patient’s name and address and the date of the invitation letter. When reply slips of acceptance or refusal have been received by the site study staff, the recruiting practice or hospital site will be informed. If no reply is received, one repeat invitation pack will be sent to potential participants.

#### Results and analysis

Interviews will be conducted by the research nurse. The interviews will be semi-structured and the questions will be open ended, neutral and sensitive to capture the experiences of older people. All interviews will be audio-recorded with the respondent’s written consent, transcribed (and edited to ensure the anonymity of the respondent). The transcripts will form the data for the formal analysis. We will use QSR NVivo data collation and management software to assist in the coding of the data. Analysis will be conducted according to the standard procedures of rigorous qualitative analysis (open and focused coding, constant comparison, deviant case analysis and memoing) [20]. We will also conduct a series of ‘data analysis clinics’ where the research team will share and exchange interpretations of emerging and key issues [21].

### SORTED C: Retrospective cohort study

#### Aim

The aim is to collect the data required to inform a sample size calculation for SORTED II, the future full study where the primary outcome will be the 4-year all-cause mortality and cardiovascular morbidity.

#### Objectives

The objectives are to collect data to inform the design of the future full study, SORTED II. In particular, they are to estimate the distribution of the time-to-death variable for treated hypothyroid patients aged 80 years and over with 4 years of follow-up and to describe a number of other variables at study entry in 2008 for the cohort (time since diagnosis of hypothyroidism, time since LT4 treatment initiation, TSH level at diagnosis, FT4 level at diagnosis, cause of death (if applicable), age, gender, smoking status, body mass index, diabetes mellitus, hypertension and any other significant co-morbidities).

#### Design

This will be a retrospective cohort of 400 treated hypothyroid patients aged 80 years or more registered in 2008 in primary care practices.

#### Sample size

A total of 400 individuals who were 80 years or more in 2008 and were being treated for hypothyroidism will be analyzed. This will provide a 95% CI for the mortality rate in the population of interest with a width of at most ±5% to inform the power calculation for the full study.

#### Sampling and participants

To identify patients for inclusion in the cohort study, the general practice will perform a database search of patient electronic records (Figure 1), which simultaneously identifies patients for SORTED A and C. Based on the information collected in the feasibility work and using an average practice list size of 7,000, we estimate that we need to recruit 20 general practices to achieve a sample size of at least 400 patients. In Northumberland, Tyne and Wear there are approximately 300 general practices of which 65 are GP research sites. The Primary Care Research Network staff will identify 20 practices to approach and will mail out the study literature. We do not anticipate a problem recruiting general practices to participate but if we cannot achieve our cohort of 400 patients from the first 20 general practices recruited, we will approach further practices.

#### Statistical analysis

As these data are being collected to inform the design of a future full study, the analyses of the data collected will be mainly descriptive, with 95% confidence intervals reported where appropriate.

#### Ethical arrangements and research governance

A favorable ethics opinion has been received from Sunderland Research Ethics Committee (REC Reference: 12/NE/0098). The conduct of this study will be in accordance with the recommendations for physicians involved in research on human subjects adopted by the 18th World Medical Assembly, Helsinki 1964 and the principles of Good Clinical Practice [22], the MHRA [19] and the *Research Governance Framework for Health and Social Care*[23].

A data monitoring and ethics committee (DMEC) has been convened to scrutinize the integrity of the trial data and associated ethical issues. The DMEC members are an independent chair, statistician and specialist endocrinologist.

The trial steering committee (TSC) will provide overall supervision for the trial on behalf of the trial sponsor, Newcastle upon Tyne Hospitals NHS Foundation Trust, and the trial funder, NIHR Research for Patient Benefit (RfPB). The committee members are an independent British Thyroid Foundation (BTF) member, the chief investigator, three co-investigators, the trial statistician and the trial manager.

The study will commence once all regulatory approvals and Trust Research and Development approvals via the NIHR Coordinated System for gaining NHS permissions (CSP) are in place for all participating sites.

## Discussion

As the UK population ages so does the prevalence of thyroid disorders. Our study group argue that serum thyroid hormone and TSH levels are not constant; with free triiodothyronine (FT3) tending to decline and median TSH levels rising in advanced age in humans. Artificially imposing lower TSH reference ranges upon the oldest old in our population may be exposing these individuals to increased all-cause mortality and cardiovascular morbidity. This study prepares the way for a definitive trial, SORTED II, to address the above hypothesis. SORTED A, B and C will test the feasibility of the RCT procedures, examine patient perspectives and describe the distribution of a number of variables to help design the definitive trial SORTED II.

Beyond SORTED II, if it is shown that older hypothyroid patients require a lower dose of LT4 and that this strategy improves morbidity and survival, then hundreds of thousands of individuals worldwide would benefit. SORTED II would provide compelling evidence to write definitive guidelines to address the management of elderly patients currently diagnosed with hypothyroidism and subclinical hypothyroidism. Furthermore, elderly individuals with mildly raised TSH levels may not require treatment at all, thus reclassifying them as ‘disease-free’.

## Trial status

The study received NHS ethics approval (REC Reference: 12/NE/0098) on 3 April 2012, NHS Trust governance approvals on 25 July 2012 and recruitment commenced on 15 October 2012. Recruitment will proceed until 31 July 2013. Results will be submitted for publication in 2014.

## Abbreviations

BTF: British Thyroid Foundation; CTX: carboxy-terminal collagen crosslinks in serum; DMEC: data monitoring and ethics committee; EQ-5D: EuroQoL 5D; FRAT: falls risk assessment tool; FT3: free triiodothyronine (thyroid hormone); FT4: free thyroxine; GP: general practitioner; HDL: high-density lipoprotein; IMP: investigational medicinal product; LT4: levothyroxine; MHRA: Medicines and Healthcare Products Regulatory Agency; NHANES III: National Health and Nutritional Estimates Survey; NIHR: National Institute for Health Research; PIC: participant identification center; PIS: participant information sheet; QoL: quality of life; RCT: randomized controlled trial; REC: research ethics committee; RfPB: Research for Patient Benefit; RVI: Royal Victoria Infirmary; serum CTX: serum collagen type-1 cross-linked C-telopeptide; ThyDQoL: underactive-thyroid-dependent quality of life questionnaire; ThySRQ: hypothyroid symptom checklist; TSC: trial steering committee; TSH: thyroid stimulating hormone; TUG: timed up and go test.

## Competing interests

The authors declare that they have no competing interests.

## Authors’ contributions

SR is the chief investigator, and conceived the study and led on protocol development and grant acquisition. SW wrote this manuscript and approved the final version for publication. SW, SP, VR, TR and LI are co-holders of the grant and contributed to protocol development, study approvals and study literature. SR and LI orchestrated drug supplies for the study. VR is the study statistician. TR leads the qualitative aspects of the study. All authors have commented upon drafts of the manuscript and have given final approval for this version.
